# Albumin-to-Alkaline Phosphatase Ratio: A Novel Prognostic Index for Hepatocellular Carcinoma

**DOI:** 10.1155/2015/564057

**Published:** 2015-02-09

**Authors:** Anthony W. H. Chan, Stephen L. Chan, Frankie K. F. Mo, Grace L. H. Wong, Vincent W. S. Wong, Yue-Sun Cheung, Henry L. Y. Chan, Winnie Yeo, Paul B. S. Lai, Ka-Fai To

**Affiliations:** ^1^Department of Anatomical and Cellular Pathology, State Key Laboratory in Oncology in South China, Prince of Wales Hospital, The Chinese University of Hong Kong, Shatin, Hong Kong; ^2^Department of Clinical Oncology, State Key Laboratory in Oncology in South China, Prince of Wales Hospital, The Chinese University of Hong Kong, Shatin, Hong Kong; ^3^Institute of Digestive Disease, Partner State Key Laboratory of Digestive Disease, The Chinese University of Hong Kong, Shatin, Hong Kong; ^4^Department of Medicine and Therapeutics, The Chinese University of Hong Kong, Shatin, Hong Kong; ^5^Division of Hepatobiliary and Pancreatic Surgery, Department of Surgery, The Chinese University of Hong Kong, Shatin, Hong Kong; ^6^Li Ka Shing Institute of Health Science, Sir Y. K. Pao Cancer Centre, The Chinese University of Hong Kong, Shatin, Hong Kong

## Abstract

Prognosis of patients with hepatocellular carcinoma (HCC) depends on both tumour extent and hepatic function reserve. Liver function test (LFT) is a basic routine blood test to evaluate hepatic function. We first analysed LFT components and their associated scores in a training cohort of 217 patients who underwent curative surgery to identify LFT parameters with high performance (discriminatory capacity, homogeneity, and monotonicity of gradient). We derived a novel index, albumin-to-alkaline phosphatase ratio (AAPR), which had the highest c-index (0.646) and *χ* ^2^ (24.774) among other liver biochemical parameters. The AAPR was an independent prognostic factor for overall and disease-free survival. The adjusted hazard ratio of death and tumour relapse was 2.36 (*P* = 0.002) and 1.85 (*P* = 0.010), respectively. The independent prognostic significance of AAPR on top of 5 commonly used and well established staging systems was further confirmed in 2 independent cohorts of patients receiving surgical resection (*n* = 256) and palliative therapy (*n* = 425). In summary, the AAPR is a novel index readily derived from a simple low-cost routine blood test and is an independent prognostic indicator for patients with HCC regardless of treatment options.

## 1. Introduction

Hepatocellular carcinoma (HCC) is the fifth commonest cancer in men and the ninth in women worldwide. It is the second most common cause of cancer-related death and responsible for nearly 746,000 deaths worldwide in 2012 [1]. Surgical resection and liver transplantation are effective curative treatment modalities for HCC. Although liver transplantation in Asia-Pacific countries has been increasing in recent years, it is still far less common than Western countries due to shortage of donor liver [2]. However, tumour relapse is a major complication of resection and affects about 70% of patients at 5 years of follow-up [3]. Even amongst patients with apparently early-stage disease (the Barcelona Clinic Liver Cancer (BCLC) stage 0/A), the 1-year disease-free survival rate is only 77%, which indicates that about one-quarter of these patients suffer from tumour relapse within the first year after resection [4]. Identification of patients at high-risk of recurrence is critical to improve the management of patients.

Several staging systems have been developed for predicting clinical outcome and guiding treatment modalities for patients with HCC. Those widely used staging systems with external validations include the American Joint Committee on Cancer (AJCC) TNM staging system [5], the BCLC system, the Cancer of the Liver Italian Program (CLIP) score [6], the Chinese University Prognostic Index (CUPI) [7], and the Japan Integrated Staging (JIS) [8]. Various serum and tissue biomarkers have been investigated to provide prognostic information. Serum alpha-fetoprotein (AFP) is the most commonly used HCC biomarker as a screening, diagnostic, and prognostic tool [9]. How is the prognostic value of a simple routine blood test, liver function test (LFT)? The clinical outcome of patients with HCC not only depends on tumour extent but also hepatic function. LFT is a basic routine blood test to evaluate hepatic function. Some of its components, including albumin, bilirubin, and alkaline phosphatase (ALP), have been shown to have prognostic significance when they are incorporated into scores reflecting liver function reserve (the Child-Pugh class and the Model for End-stage Liver Disease (MELD)) and some staging systems such as BCLC, CLIP, CUPI, and JIS [3, 6, 8, 10, 11]. However, their independent prognostic impacts have seldom been mentioned.

In the current study, we first analysed the prognostic power of LFT components in a training cohort of patients undergoing curative surgery to explore LFT parameters with high discriminatory capacities. We then derived a novel index, albumin-to-alkaline phosphatase ratio (AAPR), and examined the clinicopathological correlation and prognostic significance of the AAPR. The prognostic value of the AAPR was further validated in other 2 independent cohorts of patients receiving surgical resection and palliative therapy on top of established staging systems including AJCC, BCLC, CLIP, CUPI, and JIS systems.

## 2. Materials and Methods

### 2.1. Study Population and Variables

The training cohort and validation cohort I recruited patients who underwent curative surgical resection for primary HCC at Prince of Wales Hospital (Hong Kong, China) from January 2001 to June 2006, and July 2006 to December 2011, respectively. The validation cohort II consisted of patients who received palliative treatment for unresectable HCC at Prince of Wales Hospital from January 2007 to December 2011. The study has been approved by the institutional review board.

Baseline clinical and laboratory parameters (namely, prothrombin time and international normalized ratio (INR), liver and renal biochemistry, hepatitis B/C (HBV/HCV) serology, AFP, Child-Pugh class, etc.) were retrieved and reviewed from the hospital database. The blood tests except viral serology were taken within 1 week before treatment. The MELD was calculated based on the equation: 9.57  × (creatinine mg/dL) + 3.78 × ln(bilirubin mg/dL) + 11.2  × ln(INR) + 6.43 [10]. All patients were staged with the following 5 major staging systems: the AJCC TNM staging system (7th edition 2010), the BCLC staging system, the CLIP score, the CUPI score, and the JIS score [3, 5, 6, 8, 10, 11]. The cut-off value of AFP was 500 as reported [7]. The histological diagnosis of all patients with resected HCC was reviewed and confirmed by two pathologists (Anthony W. H. Chan and Ka-Fai To). Histological grading was based on WHO definition (well, moderately and poorly differentiated). Vascular invasion included pathologically identified gross vascular and microvascular invasions for those resectable HCCs, and radiologically detected vascular invasion for those unresectable HCCs.

All patients underwent surveillance after treatment in the clinic with regular ultrasonography and measurement of AFP according to the local practice. The duration of follow-up was defined from the date of operation (or first diagnosis for the validation cohort II) to the latest follow-up before we analysed the data or the date of death. Overall survival was defined from the time of surgery (or first diagnosis for the validation cohort II) to the time of HCC-related death. Disease-free survival was defined from the time of surgery to the time of radiological evidence of tumour recurrence or metastasis.

### 2.2. Statistical Analyses

Statistical analyses were performed using chi-square test and Fisher's exact test for difference between groups, Student's *t*-test for those between means, and Mann-Whitney test for those between medians. The Kaplan-Meier method was used to estimate the survival rates for different groups. The equivalences of the survival curves were tested by log-rank statistics. The Cox proportional hazards model with the likelihood ratio statistics was employed for univariate and multivariate survival analyses. The optimal cutoff for the AALR was determined by a receiver operating characteristic (ROC) curve. The performance of prognostic factors was evaluated by the following: (1) the differences in the survival times among patients is classified into different groups (discriminatory ability); (2) the differences in the survival times is small among patients classified into the same group (homogeneity); (3) the mean survival time for a group classified as favourable by that system is always longer than the survival times noted in less favourable groups (monotonicity of gradients) [12]. The Harrell's concordance index (c-index) was calculated by bootstrapping with 100 resamples to rank different prognostic factors according to their discriminating abilities. The likelihood ratio (LR) test was applied to evaluate the homogeneity and the monotonicity of gradient. All statistical analyses were performed by R version 3.02 (R Foundation for Statistical Computing, Vienna, Austria). A 2-tailed *P* value < 0.05 was regarded as statistically significant.

## 3. Results

### 3.1. Clinicopathological Characteristics

Table 1 lists clinical and laboratory data of the training cohort. The training cohort consisted of 217 patients, who were derived from a larger set of 242 patients after exclusion of 25 patients because of palliative surgery (*n* = 5), liver transplant (*n* = 8), recurrent HCC (*n* = 6), combined HCC-cholangiocarcinoma (*n* = 4), and incomplete laboratory data (*n* = 2). The mean age was 54.0 years; most of them were male (84.3%). One hundred and ninety patients (87.6%) had chronic HBV infection, and 59.0% had histological evidence of cirrhosis. The majority of the patients (95.4%) were classified as Child-Pugh class A. The mean MELD score was 8.6. Sixty-nine patients (31.8%) had elevated serum AFP level ≥500 *μ*g/L. The mean tumour size was 5.2 ± 3.2 cm. One hundred and sixty-six patients (76.5%) had a solitary tumour. Vascular invasion was found in 28.6% of tumours. No patient had nodal or distant metastasis. The median follow-up duration was 44.5 months (range: 0.1–160.7). At the time of analysis, 126 patients (58.1%) were alive but only 92 patients (42.4%) did not suffer from tumour relapse. The median overall and disease-free survival period was 48.0 months and 20.8 months, respectively.

### 3.2. Establishment, Clinicopathological Association, and Prognostic Significance of AAPR

In comparison among parameters evaluating liver function, c-indices and *χ* ^2^ (by LR test) of albumin and ALP were higher than those of bilirubin, alanine aminotransferase (ALT), Child-Pugh score, and MELD score (Table 2). A novel index, AAPR, was then derived from these 2 parameters with high discriminatory capacities and was calculated by dividing albumin (g/L) by ALP (IU/L). The c-index and *χ* ^2^ (by LR test) of the AAPR was highest of these parameters.

Hypoalbuminaemia (<35 g/L, lower limit of normal) and elevated ALP (>110 IU/L, upper limit of normal) were found in 18.0% and 28.1% of patients, respectively. The mean AAPR was 0.46 ± 0.20 (interquartile range: 0.33–0.54). The optimal cutoff for the AAPR was determined by a ROC curve according to overall and disease-free survival. All patients were classified into 3 groups: a high-risk AAPR (<0.23) group (8.3%), an intermediate-risk AAPR (0.23–0.68) group (82.5%), and a low-risk AAPR (>0.68) group (9.2%). The high-risk AAPR group was associated with poor prognostic features including Child-Pugh class B/C (*P* = 0.010), higher MELD score (*P* = 0.005), multiple tumours (*P* = 0.033), advanced tumour stages (AJCC (*P* = 0.026), BCLC (*P* = 0.001), CUPI (*P* = 0.001), and JIS (*P* = 0.001)). There was no association of AAPR with histological grade, tumour size, vascular invasion, and liver capsular bleach.

**Figure 1:**
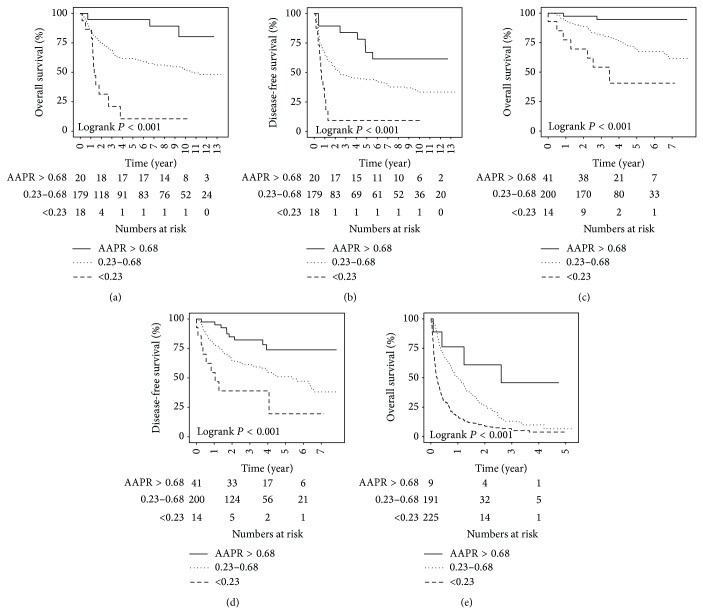
Kaplan-Meier survival plots stratified by albumin-to-alkaline phosphatase ratio in (a and b) The training cohort of patients underwent curative surgery; (c and d) the validation cohorts of patients underwent curative surgery, and (e) receiving palliative therapy.

The high-risk AAPR group was associated with poor overall survival (2-year and 5-year survival rates of 32% and 11%) compared to those of the intermediate-risk (2-year and 5-year survival rates of 76% and 61%) and low-risk (2-year and 5-year survival rates of 95% and 95%) AAPR groups (Figure 1(a)). On the univariate analysis, the following parameters were also associated with unfavourable overall survival: older age, cirrhotic background, larger tumour size, poorer histological differentiation, tumour multiplicity, the presence of vascular invasion, advanced tumour stages (AJCC, BCLC, CLIP, CUPI, and JIS), and serum AFP ≥500 *μ*g/L. The multivariate analysis on these parameters revealed that cirrhotic background, AJCC stage, and AAPR were independently associated with overall survival (Table 3). The adjusted hazard ratio (HR) of death for the AAPR was 2.36 (95% CI 1.36–4.10, *P* = 0.002).

The high-risk AAPR group was also associated with inferior disease-free survival (2-year and 5-year survival rates of 9% and 9%) compared to those of the intermediate-risk (2-year and 5-year survival rates of 53% and 44%) and low-risk (2-year and 5-year survival rates of 90% and 67%) AAPR groups (Figure 1(b)). The univariate analysis showed that unfavourable disease-free survival was associated with larger tumour size, poorer histological differentiation, tumour multiplicity, the presence of vascular invasion, advanced tumour stages (AJCC, BCLC, CLIP, CUPI, and JIS), and serum AFP ≥500 *μ*g/l. The multivariate analysis demonstrated that AJCC stage and AAPR were independent prognostic factors for disease-free survival (Table 3). The adjusted HR of tumour relapse for the AAPR was 1.85 (95% CI 1.16–2.96, *P* = 0.010).

### 3.3. Validation of Prognostic Value of AAPR

The prognostic significance of the AAPR was further validated by 2 independent and larger validation cohorts (Table 1). The validation cohort I was composed of 256 patients who underwent curative surgical resection. The clinicopathological features of patients in the validation cohort I were similar to those in the training cohort. The median follow-up duration was 38.9 months (range: 0.1–95.4). The median overall and disease-free survival period was 38.9 months and 34.1 months, respectively. All patients were stratified into 3 groups: high-risk (5.5%), intermediate-risk (78.5%), and low-risk (16.0%) AAPR groups. More patients were classified as a low-risk AAPR group because the mean AAPR of this validation cohort (0.51 ± 0.19, interquartile range: 0.38–0.63) was higher than that of the training cohort (*P* = 0.001). The high-risk AAPR group was associated with unfavourable overall survival (2-year and 5-year survival rates of 70% and 41%) compared to those of the intermediate-risk (2-year and 5-year survival rates of 89% and 69%) and low-risk (2-year and 5-year survival rates of 98% and 95%) AAPR groups (Figure 1(c)). The high-risk AAPR group was also associated with adverse disease-free survival (2-year and 5-year survival rates of 39% and 39%) compared to those of the intermediate-risk (2-year and 5-year survival rates of 65% and 51%) and low-risk (2-year and 5-year survival rates of 85% and 78%) AAPR groups (Figure 1(d)). The AAPR was an independent prognostic factor on top of all 5 staging systems in estimating overall and disease-free survival in multivariate analyses (Table 4).

The validation cohort II consisted of 425 patients with inoperable HCC receiving palliative treatments including transarterial therapy (*n* = 125), systemic chemotherapy (*n* = 99), and supportive care (*n* = 201). Patients in the validation cohort II were older in age and had higher Child-Pugh class and more advanced tumour stage, compared to those in the training cohort. The median follow-up period was 5.3 months (0.1–62.6). The median overall survival period was 5.3 months. The mean AAPR of this validation cohort (0.26 ± 0.20) was significantly lower than that of the training cohort (*P* < 0.001). All patients were distributed into 3 groups: high-risk (53.0%), intermediate-risk (44.9%), and low-risk (2.1%) AAPR groups. The high-risk AAPR group was associated with poor overall survival (6 month and 2-year survival rates of 31% and 9%) compared to those of the intermediate-risk (6 month and 2-year survival rates of 69% and 27%) and low-risk (6 month and 2-year survival rates of 76% and 61%) AAPR groups (Figure 1(e)). The AAPR was an independent prognostic factor in addition to all 5 staging systems in predicting clinical outcome in multivariate analyses (Table 4).

## 4. Discussion

Various staging systems have been developed to estimate prognosis and guide management decisions for patients with HCC. The AJCC staging system has been widely validated to provide an excellent stratification for patients who underwent surgical resection and liver transplantation [13] and is recommended by the American Hepato-Pancreato-Biliary Association for classifying surgical patients [14]. Our study reiterated this phenomenon by showing that the AJCC system has the highest c-index and *χ* ^2^ (by LR test) among surgically treated patients. However, the discriminatory capacity of the AJCC system is greatly diminished for patients with advanced unresectable HCC and poor hepatic function [5]. The discrepancy in prognostic performance of the AJCC staging system is not unexpected because the AJCC system evaluates tumour extent only, regardless of liver function reserve, which is another essential factor affecting clinical outcome of patients with HCC. Many other staging systems, such as BCLC, CLIP, CUPI, and JIS, incorporate both tumour factor and hepatic function status to provide better prognostic stratification [3, 6, 8, 11]. For patients with advanced HCC, CLIP and CUPI have been shown to be the most informative staging systems in predicting survival [15, 16]. Nevertheless, a unique universally accepted system for categorizing HCC is still not available despite a number of comparative studies and multidisciplinary consensus panel meetings [17].

LFT is a low cost and easily accessible blood test in evaluating liver function. Albumin is a protein synthesized specifically by the liver. Hypoalbuminaemia in patients with HCC is not only contributed by impaired liver function due to the underlying chronic liver disease, but also associated with a sustained systemic inflammatory response, either from the tumour itself or as a host reaction [18]. Albumin is a good indicator of hepatic protein synthetic capacity, as well as a useful marker for the host inflammatory response, which is crucial in tumorigenesis from tumour initiation to metastatic dissemination [19]. It has been integrated into several staging systems, including BCLC, CLIP, and JIS systems, to provide prognostic information for patients with HCC. ALP is a hydrolase enzyme found in all tissues throughout the whole body but is primarily present in liver, bile duct, bone, kidney, and placenta. It is an independent prognostic factor for patients with HCC [20] and is included as one of the parameters in some staging systems such as CUPI system. Our study confirmed the discriminatory power of albumin and ALP in predicting overall and disease-free survival by showing highest c-indices among other indicators of hepatic dysfunction including bilirubin, ALT, Child-Pugh score, and MELD score. However, albumin and ALP have never been put together to evaluate their combined prognostic significance. Hence we introduced a novel and simple index, AAPR. The AAPR is a powerful prognostic indicator with the highest c-index and *χ* ^2^ (by LR test) among other liver biochemical parameters. The AAPR is an independent prognostic factor for overall and disease-free survival for patients with HCC receiving curative surgery. Its prognostic significance in addition to widely used staging systems was confirmed in the validation cohort of surgically treated patients and further extended to another validation cohort of patients having palliative therapy. Stratification of patients by the AAPR into prognostically different groups allows clinicians to decide appropriate management plan for patients with HCC.

Our study has few limitations. First, our cohorts were retrospective cohorts and composed of Chinese patients only. Most were suffering from chronic HBV infection. None received other forms of curative treatments including liver transplantation and locoablative therapy. Moreover, subgroup analyses were not performed to evaluate prognostic significance of the AAPR among patients in different tumour stages because of relatively small sample size after subcategorization. Before generalization of AAPR in prognostication for HCC, it should be validated in prospective cohorts, other populations with different aetiological risk factors, patients having liver transplantation and locoablative therapy, and larger cohorts allowing subgroup analyses. The optimal cutoff for AAPR also requires external validations.

In summary, the AAPR is a novel index readily derived from a simple low-cost routine blood test and is an independent prognostic indicator for patients with HCC regardless of treatment options. It provides additional prognostic information to the widely used tumour staging systems.

## Figures and Tables

**Table 1:** Clinicopathological characteristics of 3 independent cohorts.

	Training cohort	Validation cohort I	Validation cohort II
	*n* = 217	*n* = 256	*n* = 425
*Patient factors *			
Male gender	183 (84.3%)	223 (87.1%)	378 (88.9%)
Age (years, mean ± SD)	54.0 ± 11.4	57.8 ± 10.1	60.4 ± 12.1
Hepatitis B	190 (87.6%)	208 (81.2%)	346 (81.4%)
Hepatitis C	7 (3.2%)	9 (3.5%)	28 (6.6%)
Non-B/non-C			
Alcohol liver disease	4 (1.8%)	7 (2.7%)	NA
Nonalcoholic fatty liver disease	10 (4.6%)	24 (9.4%)	NA
No known chronic liver disease	6 (2.8%)	8 (3.1%)	NA
Child-Pugh class			
A	207 (95.4%)	253 (98.8%)	266 (62.6%)
B	9 (4.1%)	3 (1.2%)	132 (31.1%)
C	1 (0.5%)	0 (0.0%)	27 (6.3%)
Liver function test			
Albumin (g/L, mean ± SD)	38.6 ± 4.7	41.0 ± 4.4	36.5 ± 15.8
Bilirubin (*μ*mol/L, mean ± SD)	12.6 ± 11.7	11.3 ± 5.8	39.0 ± 68.1
ALT (IU/L, mean ± SD)	61.6 ± 44.4	51.7 ± 39.1	80.0 ± 68.9
ALP (IU/L, mean ± SD)	97.5 ± 39.5	96.0 ± 70.7	208.6 ± 157.5
Albumin/ALP (AAPR, median)	0.45 (IQR: 0.33–0.54)	0.48 (IQR: 0.38–0.63)	0.21 (IQR: 0.13–0.33)
*Tumour factors *			
Tumour size (cm, mean ± SD)	5.2 ± 3.2	5.2 ± 3.8	9.2 ± 4.8
Histological grade			
Well-differentiated	31 (14.3%)	45 (17.6%)	NA
Moderately differentiated	150 (69.1%)	187 (73.3%)	NA
Poorly differentiated	36 (16.6%)	23 (9.0%)	NA
Multiple tumours	51 (23.5%)	57 (22.3%)	304 (71.5%)
Vascular invasion	63 (28.6%)	70 (27.3%)	163 (38.4%)
Liver capsular breach	22 (10.1%)	22 (8.6%)	NA
Lymph node metastasis	0 (0.0%)	0 (0.0%)	63 (14.8%)
Distant metastasis	0 (0.0%)	0 (0.0%)	82 (19.3%)
AJCC stage			
I	118 (54.4%)	155 (60.5%)	73 (17.2%)
II	55 (25.3%)	54 (21.1%)	95 (22.4%)
III	44 (20.3%)	47 (18.4%)	141 (33.2%)
IV	0 (0%)	0 (0%)	116 (27.3%)
BCLC stage			
0/A	155 (74.4%)	207 (80.9%)	26 (6.1%)
B	16 (7.4%)	16 (6.2%)	103 (24.2%)
C	45 (20.7%)	33 (12.9%)	266 (62.6%)
D	1 (0.5%)	0 (0%)	30 (7.1%)
CLIP score			
0	109 (50.2%)	148 (57.8%)	29 (6.8%)
1	69 (31.8%)	76 (29.7%)	71 (16.7%)
2	31 (14.3%)	27 (10.5%)	97 (22.8%)
3	6 (2.8%)	5 (2.0%)	106 (24.9%)
4/5/6	2 (0.9%)	0 (0%)	122 (28.7%)
CUPI score			
Low risk	202 (93.1%)	243 (94.9%)	167 (39.3%)
Intermediate risk	14 (6.5%)	12 (4.7%)	187 (44.0%)
High risk	1 (0.5%)	0 (0%)	71 (16.7%)
JIS score			
0	18 (8.3%)	35 (13.7%)	0 (0%)
1	106 (48.8%)	130 (50.8%)	59 (13.9%)
2	68 (31.3%)	55 (21.5%)	167 (39.3%)
3/4/5	25 (11.6%)	36 (14.1%)	199 (46.8%)
Serum AFP (*μ*g/L, median)	90.0 (IQR: 10.5–1042.0)	37.0 (IQR: 4.2–382.8)	357.0 (IQR: 21.0–15430.0)

IQR: interquartile range; NA: not available; SD: standard derivation.

**Table 2:** The discriminatory ability, homogeneity, and monotonicity of tumour stages and liver function parameters in prognostication in the training cohort.

Prognostic factor	Overall survival		Disease-free survival	
	LR test (*χ* ^2^)	*C*-index	LR test (*χ* ^2^)	*C*-index
Tumour stage				
AJCC	56.935	0.723	57.338	0.702
BCLC	33.721	0.649	36.753	0.637
CLIP	27.807	0.663	34.951	0.662
CUPI	4.214	0.531	6.694	0.538
JIS	46.120	0.708	37.194	0.669
Liver function				
Albumin	17.358	0.626	10.006	0.594
Bilirubin	7.362	0.571	5.543	0.534
ALT	4.286	0.564	5.241	0.548
ALP	14.635	0.612	13.539	0.600
Albumin/ALP (AAPR)	24.774	0.646	21.331	0.627
MELD	10.809	0.592	11.333	0.572
Child-Pugh score	9.243	0.557	5.535	0.539

**Table 3:** Univariate and multivariate analyses of prognostic factors in the training cohort.

Prognostic factor	Overall survival						Disease-free survival					
	Univariate			Multivariate			Univariate			Multivariate		
	HR	95% CI	*P* value	HR	95% CI	*P*value	HR	95% CI	*P* value	HR	95% CI	*P* value
Patient factors												
Male gender	1.726	0.895–3.332	0.104				1.635	0.952–2.806	0.075			
Age	1.021	1.002–1.040	0.027	1.015	0.996–1.036	0.126	1.012	0.996–1.029	0.134			
Hepatitis B	0.888	0.493–1.597	0.691				0.953	0.571–1.591	0.855			
Hepatitis C	1.353	0.495–3.699	0.556				1.401	0.614–3.198	0.423			
Cirrhosis	1.921	1.229–3.002	0.004	1.901	1.194–3.028	0.007	1.430	0.991–2.063	0.056			
Tumour factors												
Tumour size (cm)	1.006	1.000–1.011	0.034				1.005	1.000–1.010	0.034			
Histological grade	4.125	1.673–10.171	0.002	1.136	0.752–1.717	0.545	3.285	1.666–6.480	0.001	1.107	0.780–1.570	0.570
Multiple tumours	3.120	2.021–4.816	<0.001				2.885	1.965–4.236	<0.001			
Vascular invasion	4.371	2.862–6.676	<0.001				3.159	2.181–4.576	<0.001			
AJCC stage	2.657	2.079–3.395	<0.001	2.265	1.719–2.985	<0.001	2.400	1.937–2.972	<0.001	2.077	1.623–2.658	<0.001
BCLC stage	1.938	1.569–2.393	<0.001				1.859	1.540–2.243	<0.001			
CLIP score	1.773	1.458–2.156	<0.001				1.790	1.500–2.136	<0.001			
CUPI score	2.047	1.130–3.707	0.018				2.266	1.341–3.830	0.002			
JIS score	2.142	1.762–2.604	<0.001				1.883	1.564–2.268	<0.001			
Blood parameters												
AFP ≥500 *μ*g/L	1.893	1.244–2.883	0.003	1.465	0.916–2.341	0.111	1.985	1.384–2.847	<0.001	1.422	0.955–2.118	0.083
AAPR (>0.68/0.23–0.68/<0.23)	3.347	1.966–5.699	<0.001	2.357	1.354–4.102	0.002	2.464	1.550–3.916	<0.001	1.852	1.158–2.964	0.010
MELD	2.951	1.419–6.136	0.004	0.999	0.894–1.119	0.875	1.197	1.086–1.321	<0.001	1.068	0.964–1.184	0.208

Tumour size, multiplicity, vascular invasion, and staging systems with lower *c*-index than AJCC were excluded in the multivariate analyses to minimize multicollinearity.

**Table 4:** Multivariate analyses of combination of AAPR and established staging systems in the validation cohorts.

Prognostic factor	Validation cohort 1						Validation cohort 2		
	Overall survival			Disease-free survival			Overall survival		
	Hazard ratio	95% CI	*P* value	Hazard ratio	95% CI	*P* value	Hazard ratio	95% CI	*P* value
AJCC	2.329	1.701–3.189	<0.001	1.784	1.407–2.262	<0.001	1.474	1.322–1.643	<0.001
AAPR (>0.68/0.23–0.68/<0.23)	1.926	1.060–3.500	0.031	1.577	1.010–2.460	0.045	2.185	1.780–2.683	<0.001

BCLC	1.721	1.288–2.301	<0.001	1.567	1.246–1.971	<0.001	2.119	1.771–2.535	<0.001
AAPR (>0.68/0.23–0.68/<0.23)	2.505	1.358–4.620	0.003	1.496	1.046–2.140	0.027	1.791	1.448–2.216	<0.001

CLIP	1.641	1.220–2.207	0.001	1.398	1.109–1.764	0.005	1.678	1.539–1.829	<0.001
AAPR (>0.68/0.23–0.68/<0.23)	2.787	1.544–5.031	0.001	1.682	1.190–2.378	0.003	1.518	1.225–1.881	<0.001

CUPI	3.498	1.553–7.878	0.003	2.810	1.365–5.786	0.005	2.238	1.866–2.685	<0.001
AAPR (>0.68/0.23–0.68/<0.23)	2.500	1.327–4.709	0.005	1.622	1.135–2.318	0.008	1.306	1.024–2.685	0.0312

JIS	2.221	1.660–2.972	<0.001	1.664	1.343–2.063	<0.001	1.816	1.599–2.062	<0.001
AAPR (>0.68/0.23–0.68/<0.23)	2.144	1.173–3.921	0.013	1.449	1.013–2.073	0.042	1.644	1.325–2.040	<0.001
